# Supplementary material for: Awareness of the role of general practitioners in primary care among outpatient populations: evidence from a cross-sectional survey of tertiary hospitals in China
Source: BMJ Open. 2018 Mar 16;8(3):e020605. doi: 10.1136/bmjopen-2017-020605 (PMC5857671; doi:10.1136/bmjopen-2017-020605)
Supplement: Author's manuscript [file bmjopen-2017-020605.draft_revisions.pdf]

BMJ Open is committed to open peer review. As part of this commitment we make the peer review history of every article we publish publicly available.

When an article is published we post the peer reviewers' comments and the authors' responses online. We also post the versions of the paper that were used during peer review. These are the versions that the peer review comments apply to.

The versions of the paper that follow are the versions that were submitted during the peer review process. They are not the versions of record or the final published versions. They should not be cited or distributed as the published version of this manuscript.

BMJ Open is an open access journal and the full, final, typeset and author-corrected version of record of the manuscript is available on our site with no access controls, subscription charges or pay-per-view fees (http://bmjopen.bmj.com).

If you have any questions on BMJ Open's open peer review process please email info.bmjopen@bmj.com

BMJ Open

Limited awareness of the role of general practitioners (GPs) in primary care among outpatient populations: evidence from a cross-sectional survey of tertiary hospitals in China

Journal:	*BMJ Open*
Manuscript ID	bmjopen-2017-020605
Article Type:	Research
Date Submitted by the Author:	15-Nov-2017

Complete List of Authors:	Liu, Xiaojun; Wuhan University, School of Health Science<br>Tan, Anran; Wuhan University, School of Health Science<br>Towne, Samuel; Texas A&M University, School of Public Health<br>Hou, Zhaoxun; Wuhan University, School of Health Science<br>Mao, Zongfu; Wuhan University, School of Health Science

**Primary Subject Heading**:	Health policy
Secondary Subject Heading:	Health services research, Health policy
Keywords:	general practitioners (GPs), knowledge, influencing factors, outpatients, China

SCHOLARONE™ Manuscripts

For peer review only

Limited awareness of the role of general practitioners (GPs) in primary care among outpatient populations: evidence from a cross-sectional survey of tertiary hospitals in China

Xiaojun Liu, PhD[a,b,†], Anran Tan, MD[a,†], Samuel D. Towne Jr, PhD[c,†], Zhaoxun Hou, MD[a], Zongfu Mao, PhD[a,b,*]

[a] School of Health Sciences, Wuhan University,115# Donghu Road,Wuhan 430071, China;

[b] Global Health Institute, Wuhan University, 115# Donghu Road, Wuhan 430071, China;

[c] School of Public Health, Texas A&M University, College Station, TX 77843-1266, USA.

[†] These authors contributed equally to this work

* Correspondence to Prof. Zongfu Mao: zfmao@whu.edu.cn

Keywords: general practitioners (GPs); knowledge; influencing factors; outpatients; China

Total word count: 1867

Number of tables: 3

Number of figures: 1

For peer review only - http://bmjopen.bmj.com/site/about/guidelines.xhtml

Abstract

Objective: General practitioners (GPs) are highly underutilized in China with many patients going directly to hospitals when seeking routine care. Multiple countries around the world have successfully utilized GPs in routine care, and as such, China may benefit from the use of GPs. This study examines the status of, and factors associated with, outpatients' knowledge related to GPs in China's tertiary hospitals.

Design: This is cross-sectional survey study.

Study setting and participants: The questionnaires were completed by 565 outpatients from four tertiary hospitals in China during 2016.

Primary outcome measures: We used the logistic regression models to identify GP-related knowledge among different populations.

Results: Overall, 50.27% of respondents said they had never heard of GPs. This was also true among females (AOR=1.57, 95% CI: 1.43-2.71), older adults ($AOR_{46\text{-}65}$=1.61, 95% CI: 1.39-2.98; $AOR_{>65}$=2.01, 95% CI: 1.62-3.59), those with lower education level ($AOR_{\text{Bachelor's degree}}$=0.61, 95% CI: 0.20-0.81; $AOR_{\geq \text{Master's degree}}$=0.49, 95% CI: 0.23-0.76), rural residents (AOR=1.51, 95% CI: 1.35-2.82), and those with chronic disease ($AOR_{\text{without chronic disease}}$=0.61, 95% CI: 0.22-0.71). What's more, less than one-in-ten (9.03%) outpatients were able to accurately describe what a GP was, with less than 30% accurately describing a GP among those receiving GPs' services.

Conclusions: Outpatients who could have received less costly health services from GPs in primary medical institutions were more likely to choose costlier specialist physicians in tertiary hospitals, which is likely linked to limited knowledge about GPs. This study adds scientific rationale that to establish the national GP system more efficiently policy makers should invest in outreach efforts to improve public awareness on GPs, while at the same time conducting continued surveillance of these efforts to evaluate progress towards this goal.

For peer review only

For peer review only - http://bmjopen.bmj.com/site/about/guidelines.xhtml

Strengths and limitations of this study

1. This is the first study that represents the current status of, and factors associated with, outpatients' knowledge related to GPs in China.

2. The survey was conducted among outpatient populations from Chinese tertiary hospitals who could have received less costly health services from GPs in primary medical institutions.

3. The targeted individuals were asked to answer the questionnaires independently and anonymously to avoid the potential bias.

4. The geographic scope of the study was limited and as such may not be representative of other areas throughout China.

5. Non-response bias was not assessed, as only those agreeing to participate were included in study analyses.

For peer review only

1. Introduction

Inappropriate allocation of current health resources in China, in particular the shortage and waste in health care resources[1-3], is a critical issue that must be addressed. Medical workers in tertiary comprehensive hospitals are overwhelmed by patients, leading to long wait times for patients, and therefore increasing the potential for negative experiences among patients[4,5]. At the same time, primary care medical facilities may not be equipped with advanced medical resources, and as such, this may drive health seeking behavior towards tertiary comprehensive hospitals rather than utilization of potentially more appropriate primary care settings[6,7]. Thus, misuse of medical resources continues to be a major issue throughout China[1,4,6,8-10].

In order to achieve the goal of delivering high-quality medical resources in primary care settings in an effective way, the Chinese government is currently promoting the establishment and implementation of a hierarchical treatment system, aiming to accomplish a more efficient allocation of treatment by promoting primary care with a two-way referral mechanism, within acute and chronic patients while integrating centralized efforts and at the same time strengthening more local efforts as well. The first step in establishing a hierarchical treatment system is to develop a national general practitioner (GP) system. The general practitioners (GPs) are a critical factor in the national GP system, whose main role is to provide patients with health services in the "Integration of Six Aspects" in primary care facilities[11-13]. However, the establishment and development of the national GP system not only requires the government's relative policy guidance, but also demands the recognition from both doctors and patients to effectively implement and carry out this change.

Patients may benefit from receiving care from GPs primary care facilities with potential referral to specialist care in hospital settings as necessary. However, many seek specialist physicians in tertiary comprehensive hospitals directly, which is potentially more expensive and possibly more inconvenient given longer distances traveled by some. This phenomenon

For peer review only - http://bmjopen.bmj.com/site/about/guidelines.xhtml

may be caused by a lack of knowledge of GPs. Inadequate research exists on the role that inadequate public awareness and knowledge of GPs may play in health seeking behavior. Thus, we aimed to focus on outpatients seeking health services in top tier provincial tertiary hospitals in China. This line of inquiry can contribute to policy recommendations and accelerating the promotion of the national GP system and the hierarchical treatment system with the goal of a more efficient allocation health resources in China.

2. Materials and Methods

2.1. Participants

Outpatients with a registered physician in provincial tertiary hospitals were invited to take part in a survey, while those referred from other health service institutions were excluded.

2.2. Study procedure and data collection

We conducted a cross-sectional study using surveys in four of the top rated provincial tertiary hospitals in Jiangxi province. Survey respondents included 150 people at each hospital, with a total of 600 respondents in 2016. Convenience sampling and spatial sampling were used to collect the data. Questionnaires were given to the registered outpatients who were waiting to see a doctor in the general outpatient hall. Those who qualified were informed of the study and asked if they were willing to participate establishing informed consent. The targeted individuals were asked to answer the questionnaires independently and anonymously. Participants were interviewed by trained interviewers if they requested assistance completing the survey (e.g., outpatients who were illiterate). Lastly, completed questionnaires were checked by qualified investigators to ensure the completeness of the questionnaires with immediate follow-up with participants needing further information, as needed.

2.3. Survey tool

For peer review only

For peer review only - http://bmjopen.bmj.com/site/about/guidelines.xhtml

The survey instrument was focused on participants' knowledge of GPs. Specifically, the questionnaire inquired about (1) Social demographic characteristics including participants': sex, age, education level, average monthly income, place of residence, type of health care insurance, and chronic disease status (yes/no); (2) Participants' knowledge of GPs: consisting of six questions (Table 2).

The Cronbach's alpha coefficient of this questionnaire was 0.91, indicating that the survey tool for the present study had good internal reliability. On average, participants completed the survey in less than 3 minutes.

2.4. Quality control

Targeted individuals who completed survey questions independently, were asked to answer the questionnaires anonymously based on their own knowledge and understanding of GPs, with no mention of personal details to avoid bias due to induced prompts by the investigators. For those who could not complete survey questions independently, we employed a rigorously trained and qualified staff of investigators to interview them face-to-face by using a unified guidance language. The returned questionnaires were checked in a timely manner with incomplete surveys given back to outpatients to complete. In cases of refusal, surveys were excluded so only complete surveys were included. Double entry and logical verification of the questionnaires was done to ensure the accuracy of the data.

2.5. Statistical analysis

Statistical Package for the Social Sciences (SPSS) version 22.0 for Windows (SPSS Inc., Chicago, IL, USA) was employed to run all statistical analysis. A p-value < 0.05 was considered as statistically significant. Initial descriptive analysis summarized participants' social demographic characteristics and knowledge of GPs, with frequencies and proportions presented in Table 1, Table 2, and Figure 1. Univariate and multivariate analysis were performed by using binary logistic regression analysis to identify the main factors associated

For peer review only

For peer review only - http://bmjopen.bmj.com/site/about/guidelines.xhtml

with outpatients' knowledge related to GPs. Both the crude odds ratio (COR) and adjusted odds ratio (AOR) with associated 95% confidence intervals (95% CI) were reported in Table 3.

3. Results

3.1. Descriptions of sample demographic

As Table 1 shows, the final sample of participants was 565 with an effective survey response rate of 94.17%, of which most were men (54.34%), and urban residents (58.23%). The largest age group was 46-65 (34.69%) followed by those ages 26-45 (33.10%). Most individuals had average monthly incomes of less than 5,000 RMB with 35.22% for those making 3,000-4,999 RMB and 32.04% for those making < 3,000 RMB. Nearly 90% reported having medical insurance with 53.63% having the New Rural Cooperative Medical Care (NRCMC) and 34.51% having Medical Insurance System for Urban Residents (MISUR). Nearly a third (31.15%) had at least one chronic disease.

Table 1. Demographic information of the survey participants (n=565).

		**Frequency**	**Percentage (%)**
**Sex**	Male	307	54.34
	Female	258	45.66
**Age**	≤ 25	83	14.69
	26-45	187	33.10
	46-65	196	34.69
	> 65	99	17.52
**Education level**	≤ Junior high school	397	70.27
	Bachelor's degree	102	18.05
	≥ Master's degree	66	11.68
**AMI* (RMB)**	< 3,000	181	32.04
	3,000-4,999	199	35.22

For peer review only
For peer review only - http://bmjopen.bmj.com/site/about/guidelines.xhtml

	5,000-6,999	106	18.76
	≥ 7,000	79	13.98
**Place of residence**	City	329	58.23
	Countryside (rural)	236	41.77
**Types of health care insurance**	No insurance	67	11.86
	Yes, NRCMS*	303	53.63
	Yes, MISUR*	195	34.51
**Physical condition**	With chronic disease	176	31.15
	Without chronic disease	389	68.85

* AMI = Average monthly income; NRCMS = New rural cooperative medical system; MISUR = Medical Insurance System for Urban Residents

3.2. The status of outpatients' knowledge related to GPs

Table 2 illustrates the current status of knowledge related to GPs among outpatients from tertiary comprehensive hospitals. Overall, less than half of the subjects (49.73%) had ever heard of a GP.

Most outpatients were unable to give the correct answers on the basic conceptual knowledge towards GPs. However, there were 379 patients (67.08%) who were able to correctly identify that GPs main focus was on common and frequently occurring diseases. We also produced a figure to highlight correct answer choices where each correct answer contributes one point in three questions and the total scores is 3. The results in Figure 1 showed that there were 45 outpatients (7.96%) who scored 0 points, 394 outpatients (69.73%) who scored 1 point, 75 outpatients (13.27%) who scored 2 points, and less than one-in-ten (n=51, 9.03%) outpatients who correctly answered all questions (scored 3).

Table 2. Respondents' knowledge of the GPs.

Items	Frequency	Percentage (%)
**Have you ever heard of a GPs***		
Yes	281	49.73

For peer review only

For peer review only - http://bmjopen.bmj.com/site/about/guidelines.xhtml

No	284	50.27
**Have you received health services from GP(s)**		
Yes	153	27.08
No	66	11.68
Do not know	346	61.24
**How would you rate a GPs' technical capability**		
Good	64	11.33
Poor	149	26.37
Do not know	352	62.30
**Do you think a GP is the same as a PCP***		
Yes	516	91.33
No (correct answer)	49	8.67
**Do you think a GP is the same as a FP***		
Yes (correct answer)	88	15.58
No	477	84.42
**Do you think that GP should handle CFOD***		
Yes (correct answer)	379	67.08
No	186	32.92
**Totally**	565	100.00

* GP = General Practitioner; PCP = Primary Care Physician; FP = Family Practice; CFOD = Common and Frequently Occurring Diseases

Figure 1. Respondents' general awareness rate of knowledge towards the GPs.

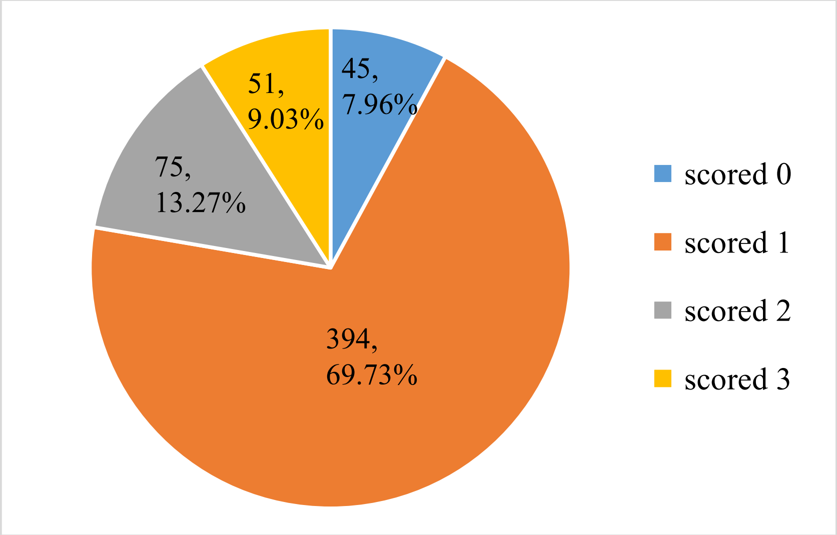

For peer review only - http://bmjopen.bmj.com/site/about/guidelines.xhtml

3.3. Analysis of knowledge towards GPs by population subgroups.

In order to distinguish the diverse knowledge levels towards GPs in different populations, we used identified whether patients had ever heard of a GP or not. Univariate and multivariate analysis were performed by using binary logistic regression models to identify the knowledge level about GPs in different groups. The results are shown in Table 3. In terms of the knowledge of GPs, both univariate and multivariate analysis showed that, female outpatients were more deficient in knowledge of GPs (OR = 1.57, 95% CI 1.43-2.71) as compared to males. When compared to younger individuals aged 25 or younger, those aged 46-65 (OR=1.61, 95% CI 1.39-2.98) and those aged 65 or above (OR=2.01, 95% CI 1.62-3.59) were more deficient in knowledge of GPs. Those with a Bachelor's degree (inclusive of having a vocational degree) were less likely to be deficient in knowledge of GPs (OR=0.61, 95% CI 0.20-0.81) and those with a Master's degree (OR=0.49, 95% CI 0.23-0.76) were also less likely to be deficient in knowledge of GPs. Rural residents were also more likely to be deficient in knowledge of GPs (OR=1.51, 95% CI 1.35-2.82) as compared to urban-dwelling adults. Further, those without chronic disease were less likely to be deficient in knowledge of GPs (OR-0.61 95% CI 0.22-0.71).

Table 3. Logistic regression analysis for participants' demographic vs. if they heard of GPs.

Demographics	No	Yes	Crude	Adjusted
	n=284 (50.27%)	n=281 (49.73%)	OR (95% CI)	OR (95% CI)
**Sex**				
Male	136 (44.30)	171 (55.70)	-	-
Female	148 (57.36)	110 (42.64)	1.69 (1.21-2.36)***	1.57 (1.43-2.71)***
**Age**				
≤ 25	35 (42.17)	48 (57.83)	-	-
26-45	86 (45.99)	101 (54.01)	1.17 (0.47-2.03)	1.02 (0.53-2.16)
46-65	105 (53.57)	91 (46.43)	1.58 (0.97-3.01)	1.61 (1.39-2.98)*
> 65	58 (58.59)	41 (41.41)	1.94 (1.73-4.15)*	2.01 (1.62-3.59)*
**Education level**				

For peer review only

For peer review only - http://bmjopen.bmj.com/site/about/guidelines.xhtml

≤ Junior high school	218 (54.91)	179 (45.09)	-	-
Bachelor's degree	41 (40.20)	61 (59.80)	0.55 (0.36-0.85)**	0.61 (0.20-0.81)**
≥ Master's degree	25 (37.88)	41 (62.12)	0.50 (0.31-0.87)**	0.49 (0.23-0.76)**
**AMI (RMB)**				
< 3,000	109 (60.22)	72 (39.78)	-	-
3,000-4,999	93 (46.73)	106 (53.27)	0.58 (0.56-0.95)**	0.52 (0.49-1.53)
5,000-6,999	51 (48.11)	55 (51.89)	0.61 (0.50-0.91)**	0.67 (0.44-1.67)
≥ 7,000	31 (39.24)	48 (60.76)	0.43 (0.34-0.93)**	0.54 (0.29-1.01)
**Place of residence**				
City	148 (44.98)	181 (55.02)	-	-
Countryside (rural)	136 (57.63)	100 (42.37)	1.66 (1.19-2.33)**	1.51 (1.35-2.82)**
**Types of health care insurance**				
No insurance	35 (52.24)	32 (47.76)	-	-
Yes, NRCMS	155 (51.16)	148 (48.84)	0.96 (0.83-1.31)	0.92 (0.76-1.73)
Yes, MISUR	94 (48.21)	101 (51.79)	0.85 (0.66-1.17)	0.86 (0.53-1.03)
**Physical condition**				
With chronic disease	108 (61.36)	68 (38.64)	-	-
Without chronic disease	176 (45.24)	213 (54.76)	0.52 (0.36-0.75)***	0.61 (0.22-0.71)***

"*" p-value <0.05, "**" p-value <0.01, "***" p-value <0.001.

4. Discussion

General practitioners may be most appropriate to care for non-emergency routine medical services, yet awareness of the role or GPs is broadly lacking. Due to China's inverted triangle of health resources allocation, the majority of patients needing health services rarely consider primary care medical facilities as their first choice, which may be a misalignment of what could be properly allocated medical treatment[1,3,4,14-17]. Therefore, many of the outpatients seeking care in tertiary comprehensive hospitals may be able to seek appropriate care from GPs in primary care health care institutions. This paper represents the current status of, and factors associated with, outpatients' knowledge related to GPs in China.

For peer review only - http://bmjopen.bmj.com/site/about/guidelines.xhtml

We believe that the establishment on the national GPs system should not only rely on administrative government departments, but also seek buy-in from medical staff, the public (the potential patients), and other key stakeholders. Our study indicates that serious deficits exist in terms of knowledge about GPs among outpatients seeking care in tertiary comprehensive hospitals. Therefore, we recommend that policy makers may use this evidence to take action by targeting awareness campaigns that highlight the role of GPs, the national GPs system, and the relative advantages of the national GPs system to the public. Specific content may cover the function of primary health care and general medical treatment in order to raise public awareness of GPs. This may take a variety of stakeholders and media including: social media (e.g., WeChat and Weibo) highlighting simple videos concerning the role and importance of GPs; medical workers who may explain relevant roles of GPs to patients; medical schools encouraging students to consider becoming GPs and/or educating them about how to discuss the importance of GPs in primary care. In terms of tailoring education and awareness campaigns to particularly at-risk groups, our findings suggest that females, older adults, those with lower education, rural residents, and those that did not have a chronic disease were most likely to have serious deficits in terms of knowledge about GPs.

Limitations

Given the target subjects in this study were already outpatients in tertiary comprehensive hospitals, the results may not necessarily generalizable to the general population in China. In addition, the geographic scope of the study was limited and as such may not be representative of other areas throughout China. Further, recall bias may have affected survey responses. Moreover, non-response bias was not assessed, as only those agreeing to participate were included in study analyses.

Conclusions

As an essential factor of the national GP system, GPs can serve as the primary provider of basic medical services for many individuals. However, Chinese outpatients from tertiary

For peer review only

For peer review only - http://bmjopen.bmj.com/site/about/guidelines.xhtml

comprehensive hospitals have limited knowledge about GPs. This is most evident among those who were female, older adults, those with lower education, rural residents, and those without chronic disease. We recommend that policy makers target investment in public education programs to raise awareness of GPs and their role. Further, continued surveillance must be carried out in order to identify whether success in achieving these targets is met over time.

Competing interests: None declared.

Funding resources: This work was performed as part of the Texas A&M University Health Science Center–Wuhan University Global Health Research Partnership. This study was also supported by an internal grant from Wuhan University (Grant Number: S2017801593). The funding body has not involved in study design, data collection, data analysis or the writing of the article.

Contributors: XL and ZM conceived the study; XL collected data and conducted the data analysis; XL, ZH, and AT drafted the paper; ZM, SDT and XL revised the manuscript. All authors read and approved the final manuscript. XL and ZM are guarantors of the paper.

Acknowledgments: We thank all the teachers, students and the research participants who took part in the data collection.

Ethical approval: This study was conducted in accordance with the Declaration of Helsinki, and the study protocol was reviewed and approved by the Institutional Review Board of Texas A&M University Health Science Center, (IRB Number: IRB2016-0285D). Local institution protocols at Wuhan University was also followed to protect participants' confidentiality.

Data sharing statement: Data are available to the corresponding author and research team only.

For peer review only - http://bmjopen.bmj.com/site/about/guidelines.xhtml

References

1. Sun J, Luo H. Evaluation on equality and efficiency of health resources allocation and health services utilization in China. *Int J Qual Health Care*, 2017; 16(1):127.

2. Jin J, Wang J, Ma X, et al. Equality of Medical Health Resource Allocation in China Based on the Gini Coefficient Method. *Iran J Public Health*, 2015; 44(4):445-457.

3. Zhang X, Zhao L, Cui Z, et al. Study on Equity and Efficiency of Health Resources and Services Based on Key Indicators in China. *Plos One*, 2015; 10(12):e0144809.

4. Pan J, Liu D, Ali S. Patient dissatisfaction in China: What matters. *Soc Sci Med*, 2015; 143:145-53.

5. Li M, Huang C, Lu X, et al. Evaluation of medical staff and patient satisfaction of Chinese hospitals and measures for improvement. *Biosci Trends*, 2015; 9(3):182.

6. Jiang, Qin, Lang, et al. Avoidable hospital admissions and resource use in China: an exploratory analysis of 2·57 million hospital admissions. *Lancet*, 2015, 386:S25-S25.

7. Cheng Z, Cai M, Tao H, et al. Efficiency and productivity measurement of rural township hospitals in China: a bootstrapping data envelopment analysis. *Bmj Open*, 2016; 6(11):e011911.

8. Liu X, Gao W, Yan H. Measuring and decomposing the inequality of maternal health services utilization in Western Rural China. *Bmc Health Serv Res*, 2014; 14(1):102.

9. Zeng J, Shi L, Zou X, et al. Rural-to-Urban Migrants' Experiences with Primary Care under Different Types of Medical Institutions in Guangzhou, China. *Plos One*, 2015; 10(10):e0140922.

10. Li K, Naganawa S, Wang K, et al. Study of the Cost-Benefit Analysis of Electronic Medical Record Systems in General Hospital in China. *J Med Syst,* 2012; 36(5):3283-3291.

For peer review only - http://bmjopen.bmj.com/site/about/guidelines.xhtml

11. Freeman A C, Sweeney K. Why general practitioners do not implement evidence: qualitative study. *BMJ*, 2001; 323(7321):1100-2.

12. Temple J. Why general practitioners do not implement evidence : Evidence seems to change frequently. *Bmj Brit Med J*, 2002; 324(7338):674-674.

13. Ping H, Luo S. A Discussion of On-the-job Training of Core General Practitioners in China. *Chinese General Practice*, 2008, 11:1132-1134. (in Chinese)

14. Tang L. The patient's anxiety before seeing a doctor and her/his hospital choice behavior in China. *Bmc Public Health*, 2012; 12(1):1-12.

15. You L M, Aiken L H, Sloane D M, et al. Hospital nursing, care quality, and patient satisfaction: cross-sectional surveys of nurses and patients in hospitals in China and Europe. *Int J Nurs Stud*, 2013; 50(2):154-161.

16. Xiong S, Luo S, Zheng X, et al. Patient and health-care provider perspectives of pregnancy-related health-care provision in Guangdong, China: a qualitative interview-based study. *Lancet Diabetes Endo*, 2016; 4:S20.

17. Song Y, Ying B, Li L. Current perspectives on China's national essential medicine system: primary care provider and patient views. *Bmc Health Serv Res*, 2015, 16(1):30.

For peer review only

For peer review only - http://bmjopen.bmj.com/site/about/guidelines.xhtml

STROBE 2007 (v4) Statement—Checklist of items that should be included in reports of *cross-sectional studies*

Section/Topic	Item #	Recommendation	Reported on page #
**Title and abstract**	1	(*a*) Indicate the study's design with a commonly used term in the title or the abstract	1
		(*b*) Provide in the abstract an informative and balanced summary of what was done and what was found	1
**Introduction**			
Background/rationale	2	Explain the scientific background and rationale for the investigation being reported	3-4
Objectives	3	State specific objectives, including any prespecified hypotheses	4
**Methods**			
Study design	4	Present key elements of study design early in the paper	4-5
Setting	5	Describe the setting, locations, and relevant dates, including periods of recruitment, exposure, follow-up, and data collection	4-5
Participants	6	(*a*) Give the eligibility criteria, and the sources and methods of selection of participants	4-5
Variables	7	Clearly define all outcomes, exposures, predictors, potential confounders, and effect modifiers. Give diagnostic criteria, if applicable	4-5
Data sources/ measurement	8*	For each variable of interest, give sources of data and details of methods of assessment (measurement). Describe comparability of assessment methods if there is more than one group	4-5
Bias	9	Describe any efforts to address potential sources of bias	4, 11
Study size	10	Explain how the study size was arrived at	4-5, Tables 1-3
Quantitative variables	11	Explain how quantitative variables were handled in the analyses. If applicable, describe which groupings were chosen and why	4-5
Statistical methods	12	(*a*) Describe all statistical methods, including those used to control for confounding	5
		(*b*) Describe any methods used to examine subgroups and interactions	4-5
		(*c*) Explain how missing data were addressed	4-5
		(*d*) If applicable, describe analytical methods taking account of sampling strategy	4-5
		(*e*) Describe any sensitivity analyses	Not reported
**Results**			

For peer review only

Participants	13*	(a) Report numbers of individuals at each stage of study—eg numbers potentially eligible, examined for eligibility, confirmed eligible, included in the study, completing follow-up, and analysed	6-9, Tables 1-3
		(b) Give reasons for non-participation at each stage	6
		(c) Consider use of a flow diagram	Not reported
Descriptive data	14*	(a) Give characteristics of study participants (eg demographic, clinical, social) and information on exposures and potential confounders	6, Table 1
		(b) Indicate number of participants with missing data for each variable of interest	Not reported
Outcome data	15*	Report numbers of outcome events or summary measures	7-8, Table 2
Main results	16	(*a*) Give unadjusted estimates and, if applicable, confounder-adjusted estimates and their precision (eg, 95% confidence interval). Make clear which confounders were adjusted for and why they were included	9-10, Table 3
		(*b*) Report category boundaries when continuous variables were categorized	Not applicable
		(*c*) If relevant, consider translating estimates of relative risk into absolute risk for a meaningful time period	Not reported
Other analyses	17	Report other analyses done—eg analyses of subgroups and interactions, and sensitivity analyses	8-9
**Discussion**			
Key results	18	Summarise key results with reference to study objectives	10-11
Limitations	19	Discuss limitations of the study, taking into account sources of potential bias or imprecision. Discuss both direction and magnitude of any potential bias	11
Interpretation	20	Give a cautious overall interpretation of results considering objectives, limitations, multiplicity of analyses, results from similar studies, and other relevant evidence	11
Generalisability	21	Discuss the generalisability (external validity) of the study results	11
**Other information**			
Funding	22	Give the source of funding and the role of the funders for the present study and, if applicable, for the original study on which the present article is based	12

*Give information separately for cases and controls in case-control studies and, if applicable, for exposed and unexposed groups in cohort and cross-sectional studies.

Note: An Explanation and Elaboration article discusses each checklist item and gives methodological background and published examples of transparent reporting. The STROBE checklist is best used in conjunction with this article (freely available on the Web sites of PLoS Medicine at http://www.plosmedicine.org/, Annals of Internal Medicine at http://www.annals.org/, and Epidemiology at http://www.epidem.com/). Information on the STROBE Initiative is available at www.strobe-statement.org.

For peer review only

BMJ Open

Limited Awareness of the role of general practitioners in primary care among outpatient populations: evidence from a cross-sectional survey of tertiary hospitals in China

Journal:	*BMJ Open*
Manuscript ID	bmjopen-2017-020605.R1
Article Type:	Research
Date Submitted by the Author:	02-Jan-2018
Complete List of Authors:	Liu, Xiaojun; Wuhan University, School of Health Science<br>Tan, Anran; Wuhan University, School of Health Science<br>Towne, Samuel; Texas A&M University, School of Public Health<br>Hou, Zhaoxun; Wuhan University, School of Health Science<br>Mao, Zongfu; Wuhan University, School of Health Science
**Primary Subject Heading**:	Health policy
Secondary Subject Heading:	Health services research, Health policy
Keywords:	general practitioners (GPs), knowledge, influencing factors, outpatients, China

SCHOLARONE™ Manuscripts

For peer review only

Limited Awareness of the role of general practitioners in primary care among outpatient populations: evidence from a cross-sectional survey of tertiary hospitals in China

Xiaojun Liu, PhD[a,b,†], Anran Tan, MD[a,†], Samuel D. Towne Jr, PhD[c,†], Zhaoxun Hou, MD[a], Zongfu Mao, PhD[a,b,*]

[a] School of Health Sciences, Wuhan University,115# Donghu Road,Wuhan 430071, China;

[b] Global Health Institute, Wuhan University, 115# Donghu Road, Wuhan 430071, China;

[c] School of Public Health, Texas A&M University, College Station, TX 77843-1266, USA.

[†] These authors contributed equally to this work

* Correspondence to Prof. Zongfu Mao: zfmao@whu.edu.cn

For peer review only

Keywords: general practitioners (GPs); knowledge; influencing factors; outpatients; China

Total word count: 1867

Number of tables: 3

Number of figures: 1

For peer review only - http://bmjopen.bmj.com/site/about/guidelines.xhtml

Abstract

Objective: General practitioners (GPs) are highly underutilized in China with many patients going directly to hospitals when seeking routine care. Multiple countries around the world have successfully utilized GPs in routine care, and as such, China may benefit from the use of GPs. This study examines the status of, and factors associated with, outpatients' knowledge related to GPs in China's tertiary hospitals.

Design: This is cross-sectional survey study.

Study setting and participants: The questionnaires were completed by 565 outpatients from four tertiary hospitals in China during 2016.

Primary outcome measures: We used the logistic regression models to identify GP-related knowledge among different populations.

Results: Overall, 50.27% of respondents said they had never heard of GPs. This was also true among females (AOR=1.57, 95% CI: 1.43-2.71), older adults ($AOR_{46\text{-}65}$=1.61, 95% CI: 1.39-2.98; $AOR_{>65}$=2.01, 95% CI: 1.62-3.59), those with lower education level ($AOR_{\text{Bachelor's degree}}$=0.61, 95% CI: 0.20-0.81; $AOR_{\geq\text{Master's degree}}$=0.49, 95% CI: 0.23-0.76), rural residents (AOR=1.51, 95% CI: 1.35-2.82), and those with chronic disease ($AOR_{\text{without chronic disease}}$=0.61, 95% CI: 0.22-0.71). What's more, less than one-in-ten (9.03%) outpatients were able to accurately describe what a GP was, with less than 30% accurately describing a GP among those receiving GPs' services.

Conclusions: Outpatients who could have received less costly health services from GPs in primary medical institutions were more likely to choose costlier specialist physicians in tertiary hospitals, which is likely linked to limited knowledge about GPs. This study adds scientific rationale that to establish the national GP system more efficiently policy makers should invest in outreach efforts to improve public awareness on GPs, while at the same time conducting continued surveillance of these efforts to evaluate progress towards this goal.

For peer review only - http://bmjopen.bmj.com/site/about/guidelines.xhtml

Strengths and limitations of this study

1. This is the first study that represents the current status of, and factors associated with, outpatients' knowledge related to GPs in China.

2. The survey was conducted among outpatient populations from Chinese tertiary hospitals who could have received less costly health services from GPs in primary medical institutions.

3. The targeted individuals were asked to answer the questionnaires independently and anonymously to avoid the potential bias.

4. The geographic scope of the study was limited and as such may not be representative of other areas throughout China.

5. Non-response bias was not assessed, as only those agreeing to participate were included in study analyses.

For peer review only

1. Introduction

Inappropriate allocation of current health resources in China, in particular the shortage and waste in health care resources[1-3], is a critical issue that must be addressed. Medical workers in tertiary comprehensive hospitals are overwhelmed by patients, leading to long wait times for patients, and therefore increasing the potential for negative experiences among patients[4,5]. At the same time, primary care medical facilities may not be equipped with advanced medical resources, and as such, this may drive health seeking behavior towards tertiary comprehensive hospitals rather than utilization of potentially more appropriate primary care settings[6,7]. Thus, misuse of medical resources continues to be a major issue throughout China[1,4,6,8-10].

In order to achieve the goal of delivering high-quality medical resources in primary care settings in an effective way, the Chinese government is currently promoting the establishment and implementation of a hierarchical treatment system, aiming to accomplish a more efficient allocation of treatment by promoting primary care with a two-way referral mechanism, within acute and chronic patients while integrating centralized efforts and at the same time strengthening more local efforts as well. The first step in establishing a hierarchical treatment system is to develop a national general practitioner (GP) system. The general practitioners (GPs) are a critical factor in the national GP system, whose main role is to provide patients with health services in the "Integration of Six Aspects" in primary care facilities[11-13]. However, the establishment and development of the national GP system not only requires the government's relative policy guidance, but also demands the recognition from both doctors and patients to effectively implement and carry out this change.

Patients may benefit from receiving care from GPs primary care facilities with potential referral to specialist care in hospital settings as necessary. However, many seek specialist physicians in tertiary comprehensive hospitals directly, which is potentially more expensive and possibly more inconvenient given longer distances traveled by some. This phenomenon

For peer review only - http://bmjopen.bmj.com/site/about/guidelines.xhtml

may be caused by a lack of knowledge of GPs. Inadequate research exists on the role that inadequate public awareness and knowledge of GPs may play in health seeking behavior. Thus, we aimed to focus on outpatients seeking health services in top tier provincial tertiary hospitals in China. This line of inquiry can contribute to policy recommendations and accelerating the promotion of the national GP system and the hierarchical treatment system with the goal of a more efficient allocation health resources in China.

2. Materials and Methods

2.1. Participants

Outpatients with a registered physician in provincial tertiary hospitals were invited to take part in a survey, while those referred from other health service institutions were excluded.

2.2. Study procedure and data collection

We conducted a cross-sectional study using surveys in four of the top rated provincial tertiary hospitals in Jiangxi province. Survey respondents included 150 people at each hospital, with a total of 600 respondents in 2016. Convenience sampling and spatial sampling were used to collect the data. Questionnaires were given to the registered outpatients who were waiting to see a doctor in the general outpatient hall. Those who qualified were informed of the study and asked if they were willing to participate establishing informed consent. The targeted individuals were asked to answer the questionnaires independently and anonymously. Participants were interviewed by trained interviewers if they requested assistance completing the survey (e.g., outpatients who were illiterate). Lastly, completed questionnaires were checked by qualified investigators to ensure the completeness of the questionnaires with immediate follow-up with participants needing further information, as needed.

2.3. Survey tool

For peer review only

For peer review only - http://bmjopen.bmj.com/site/about/guidelines.xhtml

The survey instrument was focused on participants' knowledge of GPs. Specifically, the questionnaire inquired about (1) Social demographic characteristics including (Table 1) participants': sex, age, education level, average monthly income, place of residence, type of health care insurance, and chronic disease status (yes/no); (2) Participants' knowledge of GPs: consisting of six questions (Table 2).

The Cronbach's alpha coefficient of this questionnaire was 0.91, indicating that the survey tool for the present study had good internal reliability. On average, participants completed the survey in less than 3 minutes.

2.4. Quality control

Targeted individuals who completed survey questions independently, were asked to answer the questionnaires anonymously based on their own knowledge and understanding of GPs, with no mention of personal details to avoid bias due to induced prompts by the investigators. For those who could not complete survey questions independently, we employed a rigorously trained and qualified staff of investigators to interview them face-to-face by using a unified guidance language. The returned questionnaires were checked in a timely manner with incomplete surveys given back to outpatients to complete. In cases of refusal, surveys were excluded so only complete surveys were included. Double entry and logical verification of the questionnaires was done to ensure the accuracy of the data.

2.5. Statistical analysis

Statistical Package for the Social Sciences (SPSS) version 22.0 for Windows (SPSS Inc., Chicago, IL, USA) was employed to run all statistical analysis. A p-value < 0.05 was considered as statistically significant. Initial descriptive analysis summarized participants' social demographic characteristics and knowledge of GPs, with frequencies and proportions presented in Table 1, Table 2, and Figure 1. Univariate and multivariate analysis were performed by using binary logistic regression analysis to identify the main factors associated

For peer review only - http://bmjopen.bmj.com/site/about/guidelines.xhtml

with outpatients' knowledge related to GPs. Both the crude odds ratio (COR) and adjusted odds ratio (AOR) with associated 95% confidence intervals (95% CI) were reported in Table 3.

3. Results

3.1. Descriptions of sample demographic

As Table 1 shows, the final sample of participants was 565 with an effective survey response rate of 94.17%, of which most were men (54.34%), and urban residents (58.23%). The largest age group was 46-65 (34.69%) followed by those ages 26-45 (33.10%). Most individuals had average monthly incomes of less than 5,000 RMB with 35.22% for those making 3,000-4,999 RMB and 32.04% for those making < 3,000 RMB. Nearly 90% reported having medical insurance with 53.63% having the New Rural Cooperative Medical Care (NRCMC) and 34.51% having Medical Insurance System for Urban Residents (MISUR). Nearly a third (31.15%) had at least one chronic disease.

Table 1. Demographic information of the survey participants (n=565).

		**Frequency**	**Percentage (%)**
**Sex**	Male	307	54.34
	Female	258	45.66
**Age**	≤ 25	83	14.69
	26-45	187	33.10
	46-65	196	34.69
	> 65	99	17.52
**Education level**	≤ Junior high school	397	70.27
	Bachelor's degree	102	18.05
	≥ Master's degree	66	11.68
**AMI* (RMB)**	< 3,000	181	32.04
	3,000-4,999	199	35.22

For peer review only

For peer review only - http://bmjopen.bmj.com/site/about/guidelines.xhtml

	5,000-6,999	106	18.76
	≥ 7,000	79	13.98
**Place of residence**	City	329	58.23
	Countryside (rural)	236	41.77
**Types of health care insurance**	No insurance	67	11.86
	Yes, NRCMS*	303	53.63
	Yes, MISUR*	195	34.51
**Physical condition**	With chronic disease	176	31.15
	Without chronic disease	389	68.85

* AMI = Average monthly income; NRCMS = New rural cooperative medical system; MISUR = Medical Insurance System for Urban Residents

3.2. The status of outpatients' knowledge related to GPs

Table 2 illustrates the current status of knowledge related to GPs among outpatients from tertiary comprehensive hospitals. Overall, less than half of the subjects (49.73%) had ever heard of a GP.

Most outpatients were unable to give the correct answers on the basic conceptual knowledge towards GPs. However, there were 379 patients (67.08%) who were able to correctly identify that GPs main focus was on common and frequently occurring diseases. We also produced a figure to highlight correct answer choices where each correct answer contributes one point in three questions and the total scores is 3. The results in Figure 1 showed that there were 45 outpatients (7.96%) who scored 0 points, 394 outpatients (69.73%) who scored 1 point, 75 outpatients (13.27%) who scored 2 points, and less than one-in-ten (n=51, 9.03%) outpatients who correctly answered all questions (scored 3).

Table 2. Respondents' knowledge of the GPs.

Items	Frequency	Percentage (%)
**Have you ever heard of a GPs***		
Yes	281	49.73
No	284	50.27

For peer review only

**Have you received health services from GP(s)**		
Yes	153	27.08
No	66	11.68
Do not know	346	61.24
**How would you rate a GPs' technical capability**		
Good	64	11.33
Poor	149	26.37
Do not know	352	62.30
**Do you think a GP is the same as a PCP***		
Yes	516	91.33
No (correct answer)	49	8.67
**Do you think a GP is the same as a FP***		
Yes (correct answer)	88	15.58
No	477	84.42
**Do you think that GP should handle CFOD***		
Yes (correct answer)	379	67.08
No	186	32.92
**Totally**	565	100.00

* GP = General Practitioner; PCP = Primary Care Physician; FP = Family Practice; CFOD = Common and Frequently Occurring Diseases

Figure 1. Respondents' general awareness rate of knowledge towards the GPs.

3.3. Analysis of knowledge towards GPs by population subgroups.

In order to distinguish the diverse knowledge levels towards GPs in different populations, we used identified whether patients had ever heard of a GP or not. Univariate and multivariate analysis were performed by using binary logistic regression models to identify the knowledge level about GPs in different groups. The results are shown in Table 3. In terms of the knowledge of GPs, both univariate and multivariate analysis showed that, female outpatients were more deficient in knowledge of GPs (OR = 1.57, 95% CI 1.43-2.71) as compared to males. When compared to younger individuals aged 25 or younger, those aged 46-65

For peer review only

For peer review only - http://bmjopen.bmj.com/site/about/guidelines.xhtml

(OR=1.61, 95% CI 1.39-2.98) and those aged 65 or above (OR=2.01, 95% CI 1.62-3.59) were more deficient in knowledge of GPs. Those with a Bachelor's degree (inclusive of having a vocational degree) were less likely to be deficient in knowledge of GPs (OR=0.61, 95% CI 0.20-0.81) and those with a Master's degree (OR=0.49, 95% CI 0.23-0.76) were also less likely to be deficient in knowledge of GPs. Rural residents were also more likely to be deficient in knowledge of GPs (OR=1.51, 95% CI 1.35-2.82) as compared to urban-dwelling adults. Further, those without chronic disease were less likely to be deficient in knowledge of GPs (OR-0.61 95% CI 0.22-0.71).

Table 3. Logistic regression analysis for participants' demographic vs. if they heard of GPs.

**Demographics**	**No**	**Yes**	**Crude**	**Adjusted**
	**n=284 (50.27%)**	**n=281 (49.73%)**	**OR (95% CI)**	**OR (95% CI)**
**Sex**				
Male	136 (44.30)	171 (55.70)	-	-
Female	148 (57.36)	110 (42.64)	1.69 (1.21-2.36)***	1.57 (1.43-2.71)***
**Age**				
≤ 25	35 (42.17)	48 (57.83)	-	-
26-45	86 (45.99)	101 (54.01)	1.17 (0.47-2.03)	1.02 (0.53-2.16)
46-65	105 (53.57)	91 (46.43)	1.58 (0.97-3.01)	1.61 (1.39-2.98)*
> 65	58 (58.59)	41 (41.41)	1.94 (1.73-4.15)*	2.01 (1.62-3.59)*
**Education level**				
≤ Junior high school	218 (54.91)	179 (45.09)	-	-
Bachelor's degree	41 (40.20)	61 (59.80)	0.55 (0.36-0.85)**	0.61 (0.20-0.81)**
≥ Master's degree	25 (37.88)	41 (62.12)	0.50 (0.31-0.87)**	0.49 (0.23-0.76)**
**AMI (RMB)**				
< 3,000	109 (60.22)	72 (39.78)	-	-
3,000-4,999	93 (46.73)	106 (53.27)	0.58 (0.56-0.95)**	0.52 (0.49-1.53)
5,000-6,999	51 (48.11)	55 (51.89)	0.61 (0.50-0.91)**	0.67 (0.44-1.67)
≥ 7,000	31 (39.24)	48 (60.76)	0.43 (0.34-0.93)**	0.54 (0.29-1.01)
**Place of residence**				
City	148 (44.98)	181 (55.02)	-	-

For peer review only

For peer review only - http://bmjopen.bmj.com/site/about/guidelines.xhtml

Countryside (rural)	136 (57.63)	100 (42.37)	1.66 (1.19-2.33)**	1.51 (1.35-2.82)**
**Types of health care insurance**				
No insurance	35 (52.24)	32 (47.76)	-	-
Yes, NRCMS	155 (51.16)	148 (48.84)	0.96 (0.83-1.31)	0.92 (0.76-1.73)
Yes, MISUR	94 (48.21)	101 (51.79)	0.85 (0.66-1.17)	0.86 (0.53-1.03)
**Physical condition**				
With chronic disease	108 (61.36)	68 (38.64)	-	-
Without chronic disease	176 (45.24)	213 (54.76)	0.52 (0.36-0.75)***	0.61 (0.22-0.71)***

"*" p-value <0.05, "**" p-value <0.01, "***" p-value <0.001.

4. Discussion

General practitioners may be most appropriate to care for non-emergency routine medical services, yet awareness of the role or GPs is broadly lacking. Due to China's inverted triangle of health resources allocation, the majority of patients needing health services rarely consider primary care medical facilities as their first choice, which may be a misalignment of what could be properly allocated medical treatment[1,3,4,14-17]. Therefore, many of the outpatients seeking care in tertiary comprehensive hospitals may be able to seek appropriate care from GPs in primary care health care institutions. This paper represents the current status of, and factors associated with, outpatients' knowledge related to GPs in China.

We believe that the establishment on the national GPs system should not only rely on administrative government departments, but also seek buy-in from medical staff, the public (the potential patients), and other key stakeholders. Our study indicates that serious deficits exist in terms of knowledge about GPs among outpatients seeking care in tertiary comprehensive hospitals. Therefore, we recommend that policy makers may use this evidence to take action by targeting awareness campaigns that highlight the role of GPs, the national GPs system, and the relative advantages of the national GPs system to the public. Specific content may cover the function of primary health care and general medical treatment in order to raise public awareness of GPs. This may take a variety of stakeholders and media

For peer review only

including: social media (e.g., WeChat and Weibo) highlighting simple videos concerning the role and importance of GPs; medical workers who may explain relevant roles of GPs to patients; medical schools encouraging students to consider becoming GPs and/or educating them about how to discuss the importance of GPs in primary care. In terms of tailoring education and awareness campaigns to particularly at-risk groups, our findings suggest that females, older adults, those with lower education, rural residents, and those that did not have a chronic disease were most likely to have serious deficits in terms of knowledge about GPs.

Limitations

Given the target subjects in this study were already outpatients in tertiary comprehensive hospitals, the results may not necessarily generalizable to the general population in China. In addition, the geographic scope of the study was limited and as such may not be representative of other areas throughout China. Further, recall bias may have affected survey responses. Moreover, non-response bias was not assessed, as only those agreeing to participate were included in study analyses.

Conclusions

As an essential factor of the national GP system, GPs can serve as the primary provider of basic medical services for many individuals. However, Chinese outpatients from tertiary comprehensive hospitals have limited knowledge about GPs. This is most evident among those who were female, older adults, those with lower education, rural residents, and those without chronic disease. We recommend that policy makers target investment in public education programs to raise awareness of GPs and their role. Further, continued surveillance must be carried out in order to identify whether success in achieving these targets is met over time.

Competing interests: None declared.

For peer review only - http://bmjopen.bmj.com/site/about/guidelines.xhtml

Funding resources: This work was performed as part of the Texas A&M University Health Science Center–Wuhan University Global Health Research Partnership. This study was also supported by an internal grant from Wuhan University (Grant Number: S2017801593). The funding body has not involved in study design, data collection, data analysis or the writing of the article.

Contributors: XL and ZM conceived the study; XL collected data and conducted the data analysis; XL, ZH, and AT drafted the paper; ZM, SDT and XL revised the manuscript. All authors read and approved the final manuscript. XL and ZM are guarantors of the paper.

Acknowledgments: We thank all the teachers, students and the research participants who took part in the data collection.

Ethical approval: This study was conducted in accordance with the Declaration of Helsinki, and the study protocol was reviewed and approved by the Institutional Review Board of Texas A&M University Health Science Center, (IRB Number: IRB2016-0285D). Local institution protocols at Wuhan University was also followed to protect participants' confidentiality.

Data sharing statement: Data are available to the corresponding author and research team only.

References

1. Sun J, Luo H. Evaluation on equality and efficiency of health resources allocation and health services utilization in China. *Int J Qual Health Care*, 2017; 16(1):127.

2. Jin J, Wang J, Ma X, et al. Equality of Medical Health Resource Allocation in China Based on the Gini Coefficient Method. *Iran J Public Health*, 2015; 44(4):445-457.

3. Zhang X, Zhao L, Cui Z, et al. Study on Equity and Efficiency of Health Resources and Services Based on Key Indicators in China. *Plos One*, 2015; 10(12):e0144809.

For peer review only

4. Pan J, Liu D, Ali S. Patient dissatisfaction in China: What matters. *Soc Sci Med*, 2015; 143:145-53.

5. Li M, Huang C, Lu X, et al. Evaluation of medical staff and patient satisfaction of Chinese hospitals and measures for improvement. *Biosci Trends*, 2015; 9(3):182.

6. Jiang, Qin, Lang, et al. Avoidable hospital admissions and resource use in China: an exploratory analysis of 2·57 million hospital admissions. *Lancet*, 2015, 386:S25-S25.

7. Cheng Z, Cai M, Tao H, et al. Efficiency and productivity measurement of rural township hospitals in China: a bootstrapping data envelopment analysis. *Bmj Open*, 2016; 6(11):e011911.

8. Liu X, Gao W, Yan H. Measuring and decomposing the inequality of maternal health services utilization in Western Rural China. *Bmc Health Serv Res*, 2014; 14(1):102.

9. Zeng J, Shi L, Zou X, et al. Rural-to-Urban Migrants' Experiences with Primary Care under Different Types of Medical Institutions in Guangzhou, China. *Plos One*, 2015; 10(10):e0140922.

10. Li K, Naganawa S, Wang K, et al. Study of the Cost-Benefit Analysis of Electronic Medical Record Systems in General Hospital in China. *J Med Syst,* 2012; 36(5):3283-3291.

11. Freeman A C, Sweeney K. Why general practitioners do not implement evidence: qualitative study. *BMJ*, 2001; 323(7321):1100-2.

12. Temple J. Why general practitioners do not implement evidence : Evidence seems to change frequently. *Bmj Brit Med J*, 2002; 324(7338):674-674.

13. Ping H, Luo S. A Discussion of On-the-job Training of Core General Practitioners in China. *Chinese General Practice*, 2008, 11:1132-1134. (in Chinese)

14. Tang L. The patient's anxiety before seeing a doctor and her/his hospital choice behavior in China. *Bmc Public Health*, 2012; 12(1):1-12.

For peer review only

For peer review only - http://bmjopen.bmj.com/site/about/guidelines.xhtml

15. You L M, Aiken L H, Sloane D M, et al. Hospital nursing, care quality, and patient satisfaction: cross-sectional surveys of nurses and patients in hospitals in China and Europe. *Int J Nurs Stud*, 2013; 50(2):154-161.

16. Xiong S, Luo S, Zheng X, et al. Patient and health-care provider perspectives of pregnancy-related health-care provision in Guangdong, China: a qualitative interview-based study. *Lancet Diabetes Endo*, 2016; 4:S20.

17. Song Y, Ying B, Li L. Current perspectives on China's national essential medicine system: primary care provider and patient views. *Bmc Health Serv Res*, 2015, 16(1):30.

Figure 1. Respondents' general awareness rate of knowledge towards the GPs.

For peer review only

For peer review only - http://bmjopen.bmj.com/site/about/guidelines.xhtml

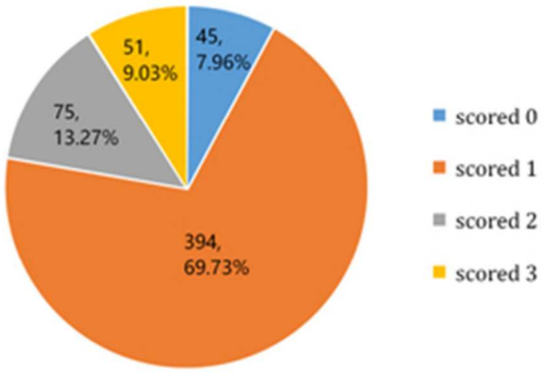

Figure 1. Respondents' general awareness rate of knowledge towards the GPs.

27x17mm (300 x 300 DPI)

For peer review only

STROBE 2007 (v4) Statement—Checklist of items that should be included in reports of *cross-sectional studies*

Section/Topic	Item #	Recommendation	Reported on page #
**Title and abstract**	1	(*a*) Indicate the study's design with a commonly used term in the title or the abstract	1
		(*b*) Provide in the abstract an informative and balanced summary of what was done and what was found	1
**Introduction**			
Background/rationale	2	Explain the scientific background and rationale for the investigation being reported	3-4
Objectives	3	State specific objectives, including any prespecified hypotheses	4
**Methods**			
Study design	4	Present key elements of study design early in the paper	4-5
Setting	5	Describe the setting, locations, and relevant dates, including periods of recruitment, exposure, follow-up, and data collection	4-5
Participants	6	(*a*) Give the eligibility criteria, and the sources and methods of selection of participants	4-5
Variables	7	Clearly define all outcomes, exposures, predictors, potential confounders, and effect modifiers. Give diagnostic criteria, if applicable	4-5
Data sources/ measurement	8*	For each variable of interest, give sources of data and details of methods of assessment (measurement). Describe comparability of assessment methods if there is more than one group	4-5
Bias	9	Describe any efforts to address potential sources of bias	4, 11
Study size	10	Explain how the study size was arrived at	4-5, Tables 1-3
Quantitative variables	11	Explain how quantitative variables were handled in the analyses. If applicable, describe which groupings were chosen and why	4-5
Statistical methods	12	(*a*) Describe all statistical methods, including those used to control for confounding	5
		(*b*) Describe any methods used to examine subgroups and interactions	4-5
		(*c*) Explain how missing data were addressed	4-5
		(*d*) If applicable, describe analytical methods taking account of sampling strategy	4-5
		(*e*) Describe any sensitivity analyses	Not reported
**Results**			

For peer review only

Participants	13*	(a) Report numbers of individuals at each stage of study—eg numbers potentially eligible, examined for eligibility, confirmed eligible, included in the study, completing follow-up, and analysed	6-9, Tables 1-3
		(b) Give reasons for non-participation at each stage	6
		(c) Consider use of a flow diagram	Not reported
Descriptive data	14*	(a) Give characteristics of study participants (eg demographic, clinical, social) and information on exposures and potential confounders	6, Table 1
		(b) Indicate number of participants with missing data for each variable of interest	Not reported
Outcome data	15*	Report numbers of outcome events or summary measures	7-8, Table 2
Main results	16	(*a*) Give unadjusted estimates and, if applicable, confounder-adjusted estimates and their precision (eg, 95% confidence interval). Make clear which confounders were adjusted for and why they were included	9-10, Table 3
		(*b*) Report category boundaries when continuous variables were categorized	Not applicable
		(*c*) If relevant, consider translating estimates of relative risk into absolute risk for a meaningful time period	Not reported
Other analyses	17	Report other analyses done—eg analyses of subgroups and interactions, and sensitivity analyses	8-9
**Discussion**			
Key results	18	Summarise key results with reference to study objectives	10-11
Limitations	19	Discuss limitations of the study, taking into account sources of potential bias or imprecision. Discuss both direction and magnitude of any potential bias	11
Interpretation	20	Give a cautious overall interpretation of results considering objectives, limitations, multiplicity of analyses, results from similar studies, and other relevant evidence	11
Generalisability	21	Discuss the generalisability (external validity) of the study results	11
**Other information**			
Funding	22	Give the source of funding and the role of the funders for the present study and, if applicable, for the original study on which the present article is based	12

*Give information separately for cases and controls in case-control studies and, if applicable, for exposed and unexposed groups in cohort and cross-sectional studies.

Note: An Explanation and Elaboration article discusses each checklist item and gives methodological background and published examples of transparent reporting. The STROBE checklist is best used in conjunction with this article (freely available on the Web sites of PLoS Medicine at http://www.plosmedicine.org/, Annals of Internal Medicine at http://www.annals.org/, and Epidemiology at http://www.epidem.com/). Information on the STROBE Initiative is available at www.strobe-statement.org.

For peer review only

BMJ Open

Awareness of the role of general practitioners in primary care among outpatient populations: evidence from a cross-sectional survey of tertiary hospitals in China

Journal:	*BMJ Open*
Manuscript ID	bmjopen-2017-020605.R2
Article Type:	Research
Date Submitted by the Author:	06-Feb-2018
Complete List of Authors:	Liu, Xiaojun; Wuhan University, School of Health Science<br>Tan, Anran; Wuhan University, School of Health Science<br>Towne, Samuel; Texas A&M University, School of Public Health<br>Hou, Zhaoxun; Wuhan University, School of Health Science<br>Mao, Zongfu; Wuhan University, School of Health Science
**Primary Subject Heading**:	Health policy
Secondary Subject Heading:	Health services research, Health policy
Keywords:	general practitioners (GPs), knowledge, influencing factors, outpatients, China

SCHOLARONE™
Manuscripts

Awareness of the role of general practitioners in primary care among outpatient populations: evidence from a cross-sectional survey of tertiary hospitals in China

Xiaojun Liu, PhD, MPH [a,b,†], Anran Tan, MD [a,†], Samuel D. Towne Jr, PhD, MPH, CPH [c,†], Zhaoxun Hou, MD [a], Zongfu Mao, PhD [a,b,*]

[a] School of Health Sciences, Wuhan University,115# Donghu Road,Wuhan 430071, China;

[b] Global Health Institute, Wuhan University, 8# South Donghu Road, Wuhan 430072, China;

[c] School of Public Health, Texas A&M University, College Station, TX 77843-1266, USA.

† These authors contributed equally to this work

* Correspondence to Prof. Zongfu Mao: zfmao@whu.edu.cn

Keywords: general practitioners (GPs); knowledge; influencing factors; outpatients; China

Total word count: 1867

Number of tables: 3

Number of figures: 1

For peer review only - http://bmjopen.bmj.com/site/about/guidelines.xhtml

Abstract

Objective: General practitioners (GPs) are highly underutilized in China with many patients going directly to hospitals when seeking routine care. Multiple countries around the world have successfully utilized GPs in routine care, and as such, China may benefit from the use of GPs. This study examines the status of, and factors associated with, knowledge related to GPs among outpatient populations from China's tertiary hospitals.

Design: This is a cross-sectional survey study.

Study setting and participants: The questionnaires were completed by 565 outpatients from four tertiary hospitals in China during 2016. Convenience sampling on different floors and throughout the outpatient building was carried out.

Primary outcome measures: We used the logistic regression models to identify GP-related knowledge among different populations.

Results: Overall, 50.27% of respondents said they had never heard of GPs. This was also true among females (AOR=1.57, 95% CI: 1.43-2.71), older adults ($AOR_{46\text{-}65}$=1.61, 95% CI: 1.39-2.98; $AOR_{>65}$=2.01, 95% CI: 1.62-3.59), those with lower education level ($AOR_{\text{Bachelor's degree}}$=0.61, 95% CI: 0.20-0.81; $AOR_{\geq\text{Master's degree}}$=0.49, 95% CI: 0.23-0.76), rural residents (AOR=1.51, 95% CI: 1.35-2.82), and those with chronic disease ($AOR_{\text{without chronic disease}}$=0.61, 95% CI: 0.22-0.71). What's more, less than one-in-ten (9.03%) outpatients were able to accurately describe what a GP was, with less than 30% accurately describing a GP among those receiving GPs' services.

Conclusions: Outpatients who could have received less costly health services from GPs in primary medical institutions were more likely to choose costlier specialist physicians in tertiary hospitals, which is likely linked to limited knowledge about GPs. Policy makers should invest in outreach efforts to improve public awareness of GPs, while at the same time conducting continued surveillance of these efforts to evaluate progress towards this goal.

For peer review only

For peer review only - http://bmjopen.bmj.com/site/about/guidelines.xhtml

Strengths and limitations of this study

1. This is the first study that represents the current status of, and factors associated with, outpatients' knowledge related to GPs in China.

2. The survey was conducted among outpatient populations from Chinese tertiary hospitals who could have received less costly health services from GPs in primary medical institutions.

3. Potential bias was reduced given the questionnaires were completed anonymously.

4. The geographic scope of the study was limited and as such may not be representative of other areas throughout China.

5. Non-response bias was not assessed, as only those agreeing to participate were included in study analyses.

For peer review only

For peer review only - http://bmjopen.bmj.com/site/about/guidelines.xhtml

1. Introduction

Inappropriate allocation of current health resources in China, in particular the shortage and waste in health care resources [1-3], is a critical issue that must be addressed. Medical workers in tertiary comprehensive hospitals are overwhelmed by patients, leading to long wait times for patients, and therefore increasing the potential for negative experiences among patients [4,5]. At the same time, primary care medical facilities may not be equipped with advanced medical resources, and as such, this may drive health seeking behavior towards tertiary comprehensive hospitals rather than utilization of potentially more appropriate primary care settings [6,7]. Thus, misuse of medical resources continues to be a major issue throughout China [1,4,6,8-10].

In order to achieve the goal of delivering high-quality medical resources in primary care settings in an effective way, the Chinese government is currently promoting the establishment and implementation of a hierarchical treatment system, aiming to accomplish a more efficient allocation of treatment by promoting primary care with a two-way referral mechanism, within acute and chronic patients while integrating centralized efforts and at the same time strengthening more local efforts as well. The first step in establishing a hierarchical treatment system is to develop a national general practitioner (GP) system. The general practitioners (GPs) are a critical factor in the national GP system, whose main role is to provide patients with health services in the "Integration of Six Aspects" in primary care facilities [11-13]. "Integration of Six Aspects" in primary care facilities refers to the community health service network system which integrates community prevention, health care, medical treatment, rehabilitation, health education and family planning technology guidance. The comprehensive function of the concept meets various requirements in health care [12-13]. However, the establishment and development of the national GP system not only requires the government's relative policy guidance, but also demands the recognition from both doctors and patients to effectively implement and carry out this change.

For peer review only - http://bmjopen.bmj.com/site/about/guidelines.xhtml

In China, general practitioners are typically referred to as 'GPs' or family practitioners (FPs). However, GPs (or FPs) in China are not the same as primary care physicians (PCPs). In China, PCPs are also referred to as 'barefoot doctors' (in Chinese 'Chijiao doctors' or 'Tongke practitioners'). PCPs (or barefoot doctors) are given basic training in western disease control and traditional Chinese medicine or ethnic medicine and typically have limited technical skills or medical capability [14]. China developed the national GP policy to provide general practice based primary care, and this has been implemented to varying extents across the country. The Chinese government continues to train qualified GPs with the goal of replacing existing PCPs (or barefoot doctors).

Patients may benefit from receiving care from GPs primary care facilities with potential referral to specialist care in hospital settings as necessary. However, many seek specialist physicians in tertiary comprehensive hospitals directly, which is potentially more expensive and possibly more inconvenient given longer distances traveled by some. This phenomenon may be caused by a lack of knowledge of GPs. Inadequate research exists on the role that inadequate public awareness and knowledge of GPs may play in health seeking behavior. Thus, we aimed to focus on outpatients seeking health services in top tier provincial tertiary hospitals in China. This line of inquiry can contribute to policy recommendations and accelerating the promotion of the national GP system and the hierarchical treatment system with the goal of a more efficient allocation health resources in China.

2. Materials and Methods

2.1. Participants

Outpatients with a registered physician in provincial tertiary hospitals were invited to take part in a survey, while those referred from other health service institutions were excluded.

2.2. Study procedure and data collection

For peer review only - http://bmjopen.bmj.com/site/about/guidelines.xhtml

We conducted a cross-sectional study using surveys in four of the top rated (e.g., largest hospitals with high levels of resources and high-quality care) provincial tertiary hospitals in Jiangxi province. Survey respondents included 150 people at each hospital, with a total of 600 respondents in 2016. Convenience sampling and spatial sampling (i.e., targeting different floors and directions to improve recruitment of potential participants) were used to collect the data. Questionnaires were given to the registered outpatients who were waiting to see a doctor in different floors and different directions of the outpatient building. Those who qualified (i.e., including those with a registered physician in a provincial tertiary hospital, while excluding those referred from another health service institutions) were informed of the study and asked if they were willing to participate establishing informed consent. The targeted individuals were asked to answer the questionnaires independently and anonymously. Participants were interviewed by trained interviewers if they requested assistance completing the survey (e.g., outpatients who were illiterate). Lastly, completed questionnaires were checked by qualified investigators (i.e., graduate students specifically trained to carryout data collection for this study) to ensure the completeness of the questionnaires with immediate follow-up with participants needing further information, as needed.

2.3. Survey tool

The questionnaire was designed based on the *Introduction to General Practice* [14], and with the help of scholars familiar with the topic with final review by clinical experts in the field (i.e., university hospital instructors of GPs). Surveys were pre-tested prior to study implementation to ensure that potential participants understood the wording and meaning of study questionnaires. The survey instrument was focused on participants' knowledge of GPs. Specifically, the questionnaire inquired about (1) Social demographic characteristics (Table 1) including participants': sex, age, education level, average monthly income, place of residence, type of health care insurance, and chronic disease status (self-reported yes/no); (2) Participants' knowledge of GPs: consisting of six questions (Table 2).

For peer review only - http://bmjopen.bmj.com/site/about/guidelines.xhtml

The Cronbach's alpha coefficient of this questionnaire was 0.91, indicating that the survey tool for the present study had good internal reliability. On average, participants completed the survey in less than 3 minutes.

2.4. Quality control

Targeted individuals who completed survey questions independently, were asked to answer the questionnaires anonymously based on their own knowledge and understanding of GPs, with no mention of personal details to avoid bias due to induced prompts by the investigators. For those who were unable to complete survey questions independently, we employed a rigorously trained and qualified staff of investigators to interview them face-to-face using a consistent and clear explanation of the purpose and significance of the study before the survey was conducted while at the same time answering questions raised by potential participants as they were encountered. The returned questionnaires were checked in a timely manner with incomplete surveys given back to outpatients to complete. In cases of refusal, surveys were excluded so only complete surveys were included. Double entry and logical verification of the questionnaires was done to ensure the accuracy of the data.

2.5. Statistical analysis

Statistical Package for the Social Sciences (SPSS) version 22.0 for Windows (SPSS Inc., Chicago, IL, USA) was employed to run all statistical analysis. A p-value < 0.05 was considered as statistically significant. Initial descriptive analysis summarized participants' social demographic characteristics and knowledge of GPs, with frequencies and proportions presented in Table 1, Table 2, and Figure 1. Univariate and multivariate analysis were performed by using binary logistic regression analysis to identify the main factors associated with outpatients' knowledge related to GPs. Both the crude odds ratio (COR) and adjusted odds ratio (AOR) with associated 95% confidence intervals (95% CI) were reported in Table 3.

For peer review only - http://bmjopen.bmj.com/site/about/guidelines.xhtml

3. Results

3.1. Descriptions of sample demographic

As Table 1 shows, the final sample of participants was 565 with an effective survey response rate of 94.17%, of which most were men (54.34%), and urban residents (58.23%). The largest age group was 46-65 (34.69%) followed by those ages 26-45 (33.10%). Most individuals had average monthly incomes of less than 5,000 RMB with 35.22% for those making 3,000-4,999 RMB and 32.04% for those making < 3,000 RMB. Nearly 90% reported having medical insurance with 53.63% having the New Rural Cooperative Medical Care (NRCMC) and 34.51% having Medical Insurance System for Urban Residents (MISUR). Nearly a third (31.15%) had at least one chronic disease.

Table 1. Demographic information of the survey participants (n=565).

		**Frequency**	**Percentage (%)**
**Sex**	Male	307	54.34
	Female	258	45.66
**Age**	≤ 25	83	14.69
	26-45	187	33.10
	46-65	196	34.69
	> 65	99	17.52
**Education level**	≤ Junior high school	397	70.27
	Bachelor's degree	102	18.05
	≥ Master's degree	66	11.68
**AMI* (RMB)**	< 3,000	181	32.04
	3,000-4,999	199	35.22
	5,000-6,999	106	18.76
	≥ 7,000	79	13.98
**Place of residence**	City	329	58.23
	Countryside (rural)	236	41.77
**Types of health care insurance**	No insurance	67	11.86

For peer review only - http://bmjopen.bmj.com/site/about/guidelines.xhtml

	Yes, NRCMS*	303	53.63
	Yes, MISUR*	195	34.51
**Physical condition**	With chronic disease	176	31.15
	Without chronic disease	389	68.85

* AMI = Average monthly income; NRCMS = New rural cooperative medical system; MISUR = Medical Insurance System for Urban Residents

3.2. The status of outpatients' knowledge related to GPs

Table 2 illustrates the current status of knowledge related to GPs among outpatients from tertiary comprehensive hospitals. Overall, less than half of the subjects (49.73%) had ever heard of a GP.

Most outpatients were unable to give the correct answers on the basic conceptual knowledge towards GPs. However, there were 379 patients (67.08%) who were able to correctly identify that GP's main focus was on common and frequently occurring diseases. We also produced a figure to highlight correct answer choices where each correct answer contributes one point in three questions and the total scores is 3. The results in Figure 1 showed that there were 45 outpatients (7.96%) who scored 0 points, 394 outpatients (69.73%) who scored 1 point, 75 outpatients (13.27%) who scored 2 points, and less than one-in-ten (n=51, 9.03%) outpatients who correctly answered all questions (scored 3).

Table 2. Respondents' knowledge of the GPs.

Items	Frequency	Percentage (%)
**Have you ever heard of a GPs***		
Yes	281	49.73
No	284	50.27
**Have you received health services from GP(s)**		
Yes	153	27.08
No	66	11.68
Do not know	346	61.24
**How would you rate a GPs' technical capability**		
Good	64	11.33

For peer review only

For peer review only - http://bmjopen.bmj.com/site/about/guidelines.xhtml

Poor	149	26.37
Do not know	352	62.30
**Do you think a GP is the same as a PCP***		
Yes	516	91.33
No (correct answer)	49	8.67
**Do you think a GP is the same as a FP***		
Yes (correct answer)	88	15.58
No	477	84.42
**Do you think that GP should handle CFOD***		
Yes (correct answer)	379	67.08
No	186	32.92
**Totally**	565	100.00

* GP = General Practitioner; PCP = Primary Care Physician; FP = Family Practice; CFOD = Common and Frequently Occurring Diseases

Figure 1. Respondents' general awareness rate of knowledge towards the GPs.

3.3. Analysis of knowledge towards GPs by population subgroups.

To assess potential differences in knowledge levels towards GPs in different populations, we identified whether patients had ever heard of a GP or not. Univariate and multivariate analysis were performed by using binary logistic regression models to identify the knowledge level about GPs in different groups. The results are shown in Table 3. In terms of the knowledge of GPs, both univariate and multivariate analysis showed that, female outpatients were more deficient in knowledge of GPs (OR = 1.57, 95% CI 1.43-2.71) as compared to males. When compared to younger individuals aged 25 or younger, those aged 46-65 (OR=1.61, 95% CI 1.39-2.98) and those aged 65 or above (OR=2.01, 95% CI 1.62-3.59) were more deficient in knowledge of GPs. Those with a Bachelor's degree (inclusive of having a vocational degree) were less likely to be deficient in knowledge of GPs (OR=0.61, 95% CI 0.20-0.81) and those with a Master's degree (OR=0.49, 95% CI 0.23-0.76) were also less likely to be deficient in knowledge of GPs. Rural residents were also more likely to be deficient in knowledge of GPs (OR=1.51, 95% CI 1.35-2.82) as compared to urban-dwelling

For peer review only - http://bmjopen.bmj.com/site/about/guidelines.xhtml

adults. Further, those without chronic disease were less likely to be deficient in knowledge of GPs (OR-0.61 95% CI 0.22-0.71).

Table 3. Knowledge about GPs among different groups

Demographics	No	Yes	Crude	Adjusted
	n=284 (50.27%)	n=281 (49.73%)	OR (95% CI)	OR (95% CI)
**Sex**				
Male	136 (44.30)	171 (55.70)	-	-
Female	148 (57.36)	110 (42.64)	1.69 (1.21-2.36)***	1.57 (1.43-2.71)***
**Age**				
≤ 25	35 (42.17)	48 (57.83)	-	-
26-45	86 (45.99)	101 (54.01)	1.17 (0.47-2.03)	1.02 (0.53-2.16)
46-65	105 (53.57)	91 (46.43)	1.58 (0.97-3.01)	1.61 (1.39-2.98)*
> 65	58 (58.59)	41 (41.41)	1.94 (1.73-4.15)*	2.01 (1.62-3.59)*
**Education level**				
≤ Junior high school	218 (54.91)	179 (45.09)	-	-
Bachelor's degree	41 (40.20)	61 (59.80)	0.55 (0.36-0.85)**	0.61 (0.20-0.81)**
≥ Master's degree	25 (37.88)	41 (62.12)	0.50 (0.31-0.87)**	0.49 (0.23-0.76)**
**AMI (RMB)**				
< 3,000	109 (60.22)	72 (39.78)	-	-
3,000-4,999	93 (46.73)	106 (53.27)	0.58 (0.56-0.95)**	0.52 (0.49-1.53)
5,000-6,999	51 (48.11)	55 (51.89)	0.61 (0.50-0.91)**	0.67 (0.44-1.67)
≥ 7,000	31 (39.24)	48 (60.76)	0.43 (0.34-0.93)**	0.54 (0.29-1.01)
**Place of residence**				
City	148 (44.98)	181 (55.02)	-	-
Countryside (rural)	136 (57.63)	100 (42.37)	1.66 (1.19-2.33)**	1.51 (1.35-2.82)**
**Types of health care insurance**				
No insurance	35 (52.24)	32 (47.76)	-	-
Yes, NRCMS	155 (51.16)	148 (48.84)	0.96 (0.83-1.31)	0.92 (0.76-1.73)
Yes, MISUR	94 (48.21)	101 (51.79)	0.85 (0.66-1.17)	0.86 (0.53-1.03)
**Physical condition**				
With chronic disease	108 (61.36)	68 (38.64)	-	-
Without chronic disease	176 (45.24)	213 (54.76)	0.52 (0.36-0.75)***	0.61 (0.22-0.71)***

"*" p-value <0.05, "**" p-value <0.01, "***" p-value <0.001.

For peer review only

For peer review only - http://bmjopen.bmj.com/site/about/guidelines.xhtml

4. Discussion

General practitioners may be most appropriate to care for non-emergency routine medical services, yet awareness of the role or GPs is broadly lacking. Due to China's inverted triangle of health resource allocation, the majority of patients in need of health services rarely consider primary care medical facilities as their first choice, which may be a misalignment of what could be properly allocated medical treatment [1,3,4,15-18]. Therefore, many of the outpatients seeking care in tertiary comprehensive hospitals may be able to seek appropriate care from GPs in primary care health care institutions. This paper represents the current status of, and factors associated with, outpatients' knowledge related to GPs in China.

According to the *2016 Statistical Yearbook of China's National Health and Family Planning Commission* [19], the total number of licensed GPs and assistant GPs was just 5.3% of the total number of physicians in China. The General Office of the State Council promulgated the *Opinion on Promoting the Construction of Hierarchical Medical Treatment System* [20], which aims to achieve a goal of having 2-3 qualified GPs for every 10,000 citizens. However, the average number of GPs per 10,000 citizens in China was just 1.27 overall, with 1.71 per 10,000 in more developed provinces in the eastern coastal areas, with 0.91 and 0.99 per 10,000 in less developed areas in central and western regions, respectively. Jiangxi Province is a typical central province in China with 0.54 GPs per 10,000 permanent residents in 2016, among which, most of the licensed GPs were specialists, leaving few who were truly trained by the standard procedures of GPs providing non-specialist care [21]. Moreover, nearly half of the GPs did not work in primary care facilities, giving way to a large gap in primary care facilities which desperately need GPs [21-22].

We believe that the establishment on the national GPs system should not only rely on administrative government departments, but also seek buy-in from medical staff, the public (the potential patients), and other key stakeholders. Our study indicates that serious deficits exist in terms of knowledge about GPs among outpatients seeking care in tertiary

For peer review only

For peer review only - http://bmjopen.bmj.com/site/about/guidelines.xhtml

comprehensive hospitals. Therefore, we recommend that policy makers may use this evidence to take action by targeting awareness campaigns that highlight the role of GPs, the national GPs system, and the relative advantages of the national GPs system to the public.

Specific content may cover the function of primary health care and general medical treatment in order to raise public awareness of GPs. This may take a variety of stakeholders and media including: social media (e.g., WeChat and Weibo) highlighting simple videos concerning the role and importance of GPs; medical workers who may explain relevant roles of GPs to patients; medical schools encouraging students to consider becoming GPs and/or educating them about how to discuss the importance of GPs in primary care. In terms of tailoring education and awareness campaigns to particularly at-risk groups, our findings suggest that females, older adults, those with lower education, rural residents, and those that did not have a chronic disease were most likely to have serious deficits in terms of knowledge about GPs.

Limitations

Given the target subjects in this study were already outpatients in tertiary comprehensive hospitals, the results may not necessarily generalizable to the general population in China. In addition, the geographic scope of the study was limited and as such may not be representative of other areas throughout China. Further, recall bias may have affected survey responses. Moreover, non-response bias was not assessed, as only those agreeing to participate were included in study analyses. Given the pragmatic nature of data collection in hospital settings, we did not track how many surveys were done through face-to-face interviews versus those where individuals completed the surveys by themselves or those where certain questions were missing, and participants were asked to complete missing items.

Conclusions

For peer review only - http://bmjopen.bmj.com/site/about/guidelines.xhtml

As an essential factor of the national GP system, GPs can serve as the primary provider of basic medical services for many individuals. However, Chinese outpatients from tertiary comprehensive hospitals have limited knowledge about GPs. This is most evident among those who were female, older adults, those with lower education, rural residents, and those without chronic disease. We recommend that policy makers target investment in public education programs to raise awareness of GPs and their role. Further, continued surveillance must be carried out in order to identify whether success in achieving these targets is met over time.

Competing interests: None declared.

Funding resources: This work was performed as part of the Texas A&M University Health Science Center–Wuhan University Global Health Research Partnership. This study was also supported by an internal grant from Wuhan University (Grant Number: S2017801593). The funding body has not involved in study design, data collection, data analysis or the writing of the article.

Contributors: XL and ZM conceived the study; XL collected data and conducted the data analysis; XL, ZH, and AT drafted the paper; ZM, SDT and XL revised the manuscript. All authors read and approved the final manuscript. XL and ZM are guarantors of the paper.

Acknowledgments: We thank all the teachers, students and the research participants who took part in the data collection.

Ethical approval: This study was conducted in accordance with the Declaration of Helsinki. Local institutional protocols at Wuhan University were also followed to protect participants' confidentiality, with ethics approval granted by Wuhan University's School of Public Health (IRB Number: J-17-2016). Informed consent was obtained from all participants. Hospitals are considered public places in China, and approval from the ethics committees of the hospitals

For peer review only

For peer review only - http://bmjopen.bmj.com/site/about/guidelines.xhtml

275 was not required. The Institutional Review Board of Texas A&M University (IRB Number:
276 IRB2016-0285D) also reviewed and approved of study protocols.

277 **Data sharing statement:** Data are available upon request from the corresponding author.
278 Data requesters are required to provide their research objective, design, and ethical approval
279 documents.

280 **References**

281 1. Sun J, Luo H. Evaluation on equality and efficiency of health resources allocation and
282 health services utilization in China. *Int J Qual Health Care*, 2017; 16(1):127.
283 2. Jin J, Wang J, Ma X, et al. Equality of Medical Health Resource Allocation in China Based
284 on the Gini Coefficient Method. *Iran J Public Health*, 2015; 44(4):445-457.

285 3. Zhang X, Zhao L, Cui Z, et al. Study on Equity and Efficiency of Health Resources and
286 Services Based on Key Indicators in China. *Plos One*, 2015; 10(12):e0144809.

287 4. Pan J, Liu D, Ali S. Patient dissatisfaction in China: What matters. *Soc Sci Med*, 2015;
288 143:145-53.

289 5. Li M, Huang C, Lu X, et al. Evaluation of medical staff and patient satisfaction of Chinese
290 hospitals and measures for improvement. *Biosci Trends*, 2015; 9(3):182.

291 6. Jiang, Qin, Lang, et al. Avoidable hospital admissions and resource use in China: an
292 exploratory analysis of 2·57 million hospital admissions. *Lancet*, 2015, 386:S25-S25.

293 7. Cheng Z, Cai M, Tao H, et al. Efficiency and productivity measurement of rural township
294 hospitals in China: a bootstrapping data envelopment analysis. *Bmj Open*, 2016;
295 6(11):e011911.

296 8. Liu X, Gao W, Yan H. Measuring and decomposing the inequality of maternal health
297 services utilization in Western Rural China. *Bmc Health Serv Res*, 2014; 14(1):102.

For peer review only - http://bmjopen.bmj.com/site/about/guidelines.xhtml

9. Zeng J, Shi L, Zou X, et al. Rural-to-Urban Migrants' Experiences with Primary Care under Different Types of Medical Institutions in Guangzhou, China. *Plos One*, 2015; 10(10):e0140922.

10. Li K, Naganawa S, Wang K, et al. Study of the Cost-Benefit Analysis of Electronic Medical Record Systems in General Hospital in China. *J Med Syst,* 2012; 36(5):3283-3291.

11. Freeman A C, Sweeney K. Why general practitioners do not implement evidence: qualitative study. *BMJ*, 2001; 323(7321):1100-2.

12. Temple J. Why general practitioners do not implement evidence : Evidence seems to change frequently. *Bmj Brit Med J*, 2002; 324(7338):674-674.

13. Ping H, Luo S. A Discussion of On-the-job Training of Core General Practitioners in China. *Chinese General Practice*, 2008, 11:1132-1134. (in Chinese)

14. Zhu S.Z. Introduction to General Practice (Fourth Edition). Beijing: People's Medical Publishing House, 2013. (in Chinese)

15. Tang L. The patient's anxiety before seeing a doctor and her/his hospital choice behavior in China. *Bmc Public Health*, 2012; 12(1):1-12.

16. You L M, Aiken L H, Sloane D M, et al. Hospital nursing, care quality, and patient satisfaction: cross-sectional surveys of nurses and patients in hospitals in China and Europe. *Int J Nurs Stud*, 2013; 50(2):154-161.

17. Xiong S, Luo S, Zheng X, et al. Patient and health-care provider perspectives of pregnancy-related health-care provision in Guangdong, China: a qualitative interview-based study. *Lancet Diabetes Endo*, 2016; 4:S20.

18. Song Y, Ying B, Li L. Current perspectives on China's national essential medicine system: primary care provider and patient views. *Bmc Health Serv Res*, 2015, 16(1):30.

For peer review only - http://bmjopen.bmj.com/site/about/guidelines.xhtml

19. National Health and Family Planning Commission of People's Republic of China. 2016 Statistical Yearbook of China's National Health and Family Planning Commission. Peking Union Medical College Press: Beijing, China, 2016. (In Chinese)

20. The General Office of the State Council of People's Republic of China. Available online: http://www.gov.cn/zhengce/content/2015-09/11/content_10158.htm (accessed on 16 December 2017) (In Chinese)

21. Health and Family Planning Commission of Jiangxi Province. Analysis report on the development of health and family planning in Jiangxi Province in 2016. Available online: http://www.jxwst.gov.cn/doc/2017/08/23/106893.shtml (accessed on 16 December 2017) (In Chinese)

22. Liu X, Wu M, Zhang J, Jiang X, Liu D, Wu S, Chen M, Hu Y, Yuan Z. Research on the current situation and policy of general practice in primary health care institutions in China. Chinese J. Health Mana., 34(1), 47-50. (In Chinese)

For peer review only

For peer review only - http://bmjopen.bmj.com/site/about/guidelines.xhtml

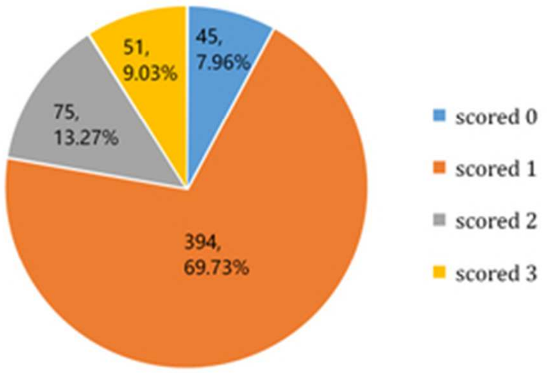

Figure 1. Respondents' general awareness rate of knowledge towards the GPs.

27x17mm (300 x 300 DPI)

For peer review only

STROBE 2007 (v4) Statement—Checklist of items that should be included in reports of *cross-sectional studies*

Section/Topic	Item #	Recommendation	Reported on page #
**Title and abstract**	1	(*a*) Indicate the study's design with a commonly used term in the title or the abstract	1
		(*b*) Provide in the abstract an informative and balanced summary of what was done and what was found	1
**Introduction**			
Background/rationale	2	Explain the scientific background and rationale for the investigation being reported	3-4
Objectives	3	State specific objectives, including any prespecified hypotheses	4
**Methods**			
Study design	4	Present key elements of study design early in the paper	4-5
Setting	5	Describe the setting, locations, and relevant dates, including periods of recruitment, exposure, follow-up, and data collection	4-5
Participants	6	(*a*) Give the eligibility criteria, and the sources and methods of selection of participants	4-5
Variables	7	Clearly define all outcomes, exposures, predictors, potential confounders, and effect modifiers. Give diagnostic criteria, if applicable	4-5
Data sources/ measurement	8*	For each variable of interest, give sources of data and details of methods of assessment (measurement). Describe comparability of assessment methods if there is more than one group	4-5
Bias	9	Describe any efforts to address potential sources of bias	4, 11
Study size	10	Explain how the study size was arrived at	4-5, Tables 1-3
Quantitative variables	11	Explain how quantitative variables were handled in the analyses. If applicable, describe which groupings were chosen and why	4-5
Statistical methods	12	(*a*) Describe all statistical methods, including those used to control for confounding	5
		(*b*) Describe any methods used to examine subgroups and interactions	4-5
		(*c*) Explain how missing data were addressed	4-5
		(*d*) If applicable, describe analytical methods taking account of sampling strategy	4-5
		(*e*) Describe any sensitivity analyses	Not reported
**Results**			

For peer review only

Participants	13*	(a) Report numbers of individuals at each stage of study—eg numbers potentially eligible, examined for eligibility, confirmed eligible, included in the study, completing follow-up, and analysed	6-9, Tables 1-3
		(b) Give reasons for non-participation at each stage	6
		(c) Consider use of a flow diagram	Not reported
Descriptive data	14*	(a) Give characteristics of study participants (eg demographic, clinical, social) and information on exposures and potential confounders	6, Table 1
		(b) Indicate number of participants with missing data for each variable of interest	Not reported
Outcome data	15*	Report numbers of outcome events or summary measures	7-8, Table 2
Main results	16	(*a*) Give unadjusted estimates and, if applicable, confounder-adjusted estimates and their precision (eg, 95% confidence interval). Make clear which confounders were adjusted for and why they were included	9-10, Table 3
		(*b*) Report category boundaries when continuous variables were categorized	Not applicable
		(*c*) If relevant, consider translating estimates of relative risk into absolute risk for a meaningful time period	Not reported
Other analyses	17	Report other analyses done—eg analyses of subgroups and interactions, and sensitivity analyses	8-9
**Discussion**			
Key results	18	Summarise key results with reference to study objectives	10-11
Limitations	19	Discuss limitations of the study, taking into account sources of potential bias or imprecision. Discuss both direction and magnitude of any potential bias	11
Interpretation	20	Give a cautious overall interpretation of results considering objectives, limitations, multiplicity of analyses, results from similar studies, and other relevant evidence	11
Generalisability	21	Discuss the generalisability (external validity) of the study results	11
**Other information**			
Funding	22	Give the source of funding and the role of the funders for the present study and, if applicable, for the original study on which the present article is based	12

*Give information separately for cases and controls in case-control studies and, if applicable, for exposed and unexposed groups in cohort and cross-sectional studies.

Note: An Explanation and Elaboration article discusses each checklist item and gives methodological background and published examples of transparent reporting. The STROBE checklist is best used in conjunction with this article (freely available on the Web sites of PLoS Medicine at http://www.plosmedicine.org/, Annals of Internal Medicine at http://www.annals.org/, and Epidemiology at http://www.epidem.com/). Information on the STROBE Initiative is available at www.strobe-statement.org.

For peer review only
